# Neuromodulation of brain power topography and network topology by prefrontal transcranial photobiomodulation

**DOI:** 10.1088/1741-2552/ac9ede

**Published:** 2022-11-17

**Authors:** Sadra Shahdadian, Xinlong Wang, Hashini Wanniarachchi, Akhil Chaudhari, Nghi Cong Dung Truong, Hanli Liu

**Affiliations:** 1 Department of Bioengineering, The University of Texas at Arlington, Arlington, TX 76019, United States of America

**Keywords:** transcranial photobiomodulation, tPBM, EEG, functional connectivity, imaginary part of coherence, graph theory

## Abstract

*Objective.* Transcranial photobiomodulation (tPBM) has shown promising benefits, including cognitive improvement, in healthy humans and in patients with Alzheimer’s disease. In this study, we aimed to identify key cortical regions that present significant changes caused by tPBM in the electroencephalogram (EEG) oscillation powers and functional connectivity in the healthy human brain. *Approach*. A 64-channel EEG was recorded from 45 healthy participants during a 13 min period consisting of a 2 min baseline, 8 min tPBM/sham intervention, and 3 min recovery. After pre-processing and normalizing the EEG data at the five EEG rhythms, cluster-based permutation tests were performed for multiple comparisons of spectral power topographies, followed by graph-theory analysis as a topological approach for quantification of brain connectivity metrics at global and nodal/cluster levels. *Main results*. EEG power enhancement was observed in clusters of channels over the frontoparietal regions in the alpha band and the centroparietal regions in the beta band. The global measures of the network revealed a reduction in synchronization, global efficiency, and small-worldness of beta band connectivity, implying an enhancement of brain network complexity. In addition, in the beta band, nodal graphical analysis demonstrated significant increases in local information integration and centrality over the frontal clusters, accompanied by a decrease in segregation over the bilateral frontal, left parietal, and left occipital regions. *Significance.* Frontal tPBM increased EEG alpha and beta powers in the frontal-central-parietal regions, enhanced the complexity of the global beta-wave brain network, and augmented local information flow and integration of beta oscillations across prefrontal cortical regions. This study sheds light on the potential link between electrophysiological effects and human cognitive improvement induced by tPBM.

## 1. Introduction

Transcranial photobiomodulation (tPBM) is a non-invasive neuromodulation technique that delivers near-infrared (NIR) light to the human brain using lasers or light-emitting diode (LED) clusters [1–4]. Recent studies have demonstrated the promising effects of tPBM in the treatment of traumatic brain injuries [4–8], psychiatric or neurological disorders [9–12], and enhanced cognitive performance in normal humans [13–18]. To better examine the underlying mechanism of tPBM, neurophysiological measurements of the human brain were performed noninvasively from human controls using optical spectroscopy before, during, and after prefrontal tPBM. These measures quantified tPBM-induced increases in mitochondrial metabolism (i.e. Δ[CCO]) and hemodynamic oxygenation (i.e. oxygenated hemoglobin (Δ[HbO])) [19–21]. It was also shown that the increases in both Δ[CCO] and Δ[HbO] were not caused by thermal effects of tPBM [22], hardware-related noise, or drift [23, 24]. All these published reports strongly support that tPBM facilitates the photo-oxidization of mitochondrial CCO to boost the cellular metabolism of neurons [2, 25–27]. The enhancement of mitochondrial activity is expected to increase cerebral oxygen demand, blood flow, and blood oxygenation, as reported in recent literature [19, 28–30]. One of these studies suggested the modulation of vasomotion in the cerebral vasculature stimulated by nitric-oxide release as another effect of tPBM [30]. Furthermore, several recent studies showed the ability of tPBM of reversing the adverse effect of aging on oxidative energy metabolism in rats [31] and improving the flow of cerebrospinal fluid at sleep in humans [32, 33].

However, the electrophysiological response of the human brain to tPBM is not well studied and understood. Table 1 summarizes the recently published articles that reported scalp electroencephalography (EEG) responses to tPBM with the respective measurement and analysis parameters. All studies utilized either laser or LED clusters of NIR light and recorded the electrophysiological responses using a 19-channel or 64-channel EEG system. The table also lists two major analysis methods, namely, power spectral density (PSD) analysis and graph theory analysis (GTA). Graph theory quantifies the specific features of network architecture (topology). The outcome of GTA can provide information on the anatomical localization of areas responding to given stimuli or human brain functional connectivity [34].

**Table 1.** List of references that reported EEG responses to tPBM with related measurement and analysis parameters.

References	Authors	Source of tPBM	Location of tPBM	# of Channels	PSD analysis	Graph-theory based connectivity analysis
[35]	Berman *et al* 2017	Multiple LED clusters (1070 nm)	Whole head	19	No. It was based on qEEG analysis	No
[36]	Ghaderi *et al* 2021	1 cluster of LED (850 nm)	Right forehead	19	No.	Yes; changes in connectivity within each of or between two hemispheres
[37]	Spera *et al*	4 clusters of LED (830 nm)	Bilateral frontal	19	Yes, with topography	No
[16]	Vargas *et al* 2017	Laser (1064 nm)	Right forehead	19	Yes, but no topography	No
[38]	Wang *et al* 2021	Laser (1064 nm)	Right forehead	64	Yes, with topography	No
[39]	Wang *et al* 2022	Laser (1064 nm)	Right forehead	64	No. It was based on singular value decompensation	No
[40]	Zomorrodi *et al* 2019	3 clusters of LED (810 nm)	3 default mode locations	19	Yes, with topography	Yes; changes in global connectivity parameters only
	Current study	Laser (1064 nm)	Right forehead	64	Yes, with topography	Yes; changes of connectivity in global network metrics and 10 nodal regions

PSD is the most common method for analysing EEG data and provides absolute power spectra of EEG in the frequency range of 0.5–70 Hz. Frequency-dependent PSD values facilitate a better understanding of impacts of external stimuli, cognitive decline, and certain brain disorders in the human brain [41–46]. Multi-channel EEG requires a multivariate statistical analysis for spatial identification where significant alterations of EEG signals occur. Accordingly, cluster-based permutation testing (CBPT) is an established method for minimizing type-I errors in EEG multivariate analysis [47, 48]. In this study, we utilized CBPT to identify topographical clusters of EEG channels on the human scalp template, where tPBM significantly altered frequency-specific EEG powers.

GTA enables to characterize functional networks in the human brain [49]. In this method, a network is a mathematical representation of a real-world complex system and is defined by the composition of nodes (vertices) and links (edges) between pairs of nodes. The outcome of GTA measures represents the functional integration, segregation, and centrality of the network, all of which can topologically characterize changes in brain functional connectivity in global and nodal regions [50–52]. When GTA is used to analyze EEG data, the scalp locations of the EEG electrodes represent the network nodes, and the links among the electrodes represent the functional connections between these nodes [51].

Both PSD and GTA-derived parameters can provide instructive information on functional brain networks in the resting state or under external neuromodulation. Because multi-channel EEG signals contain rich information in the temporal, spectral, and spatial domains, these pieces of information can be grouped temporally and/or spectrally to visualize brain activation and networks in topographical clusters and regions [53, 54].

Although existing publications (see table 1) reported that tPBM enables alterations in EEG PSDs, different tPBM protocols yielded sparse, incomparable findings. For example, only two studies showed 64-channel EEG responses to tPBM [38, 39], without quantification of tPBM-induced topological alterations in brain connectivity. Thus, the focus of this study was to investigate tPBM-induced modulations of EEG functional connectivity by performing GTA on a 64-channel EEG, followed by the quantification of changes in brain connectivity in the global network and 10 nodal regions.

## 2. Materials and methods

### 2.1. Participants

A total of 49 healthy human subjects (30 men and 19 women; 26 ± 8.8 years of age) were enrolled from the local community of the University of Texas at Arlington. The experimental protocol was approved by the Institutional Review Board of the University of Texas at Arlington and complied with all applicable federal and National Institute of Health (NIH) guidelines. Written informed consent was obtained from each participant before starting the first measurement. Four participants were removed from the dataset because of self-reported or observed sleepiness during the measurement, resulting in 45 participants being included for data analysis. The participants were instructed to refrain from consuming caffeinated drinks for at least three hours before each experiment.

### 2.2. Experimental setup and protocol

In this study, tPBM and sham experiments were performed using a continuous-wave laser at 1064 nm (Model CG-5000 Laser, Cell Gen. Therapeutics LLC, Dallas, TX, USA), which was cleared by the Food and Drug Administration. The laser was delivered to each participant’s right forehead, with an aperture of 4.2 cm in diameter and a period of 8 min. A sham experiment was performed with the laser power set to be 0.1 W and the laser aperture covered with a black cap. Table 2 lists the key parameters of light delivery used in the study. Note that a penetration rate of 1%–2% was used to estimate light deposition on the cortex [55, 56].

**Table 2.** Parameters for 8 min tPBM and sham by a 4.2 cm diameter laser at 1064 nm over the right forehead.

Stimulation	Irradiance (mW cm^−2^)	Dose density (J cm^−2^)	Total Dose (J)
tPBM on scalp	250	120	1662
tPBM on cortex*	∼2.5	∼1.20	∼16.62
Sham	0	0	0

Our tPBM protocol was designed based on previous studies [3, 13–16, 57]. The reason for using the 8 min tPBM period was because it was effective in significantly improving human cognition. The estimated dose density delivered to the human cortex was within the range of positive or photo-stimulatory responses (0.001–10 J cm^−2^) [26, 58].

Participants wore protective goggles throughout the experiment. EEG data were collected using a 64-channel EEG instrument (Biosemi, Netherlands). Each subject wore an EEG cap according to the standard 10–10 EEG electrode placement [59]. Electrode gel was used for keeping the electrode impedances below a pre-set threshold (<10 kΩ). The recorded EEG time series were directed to a computer. We followed the EEG measurement procedures described in [38, 60].

**Figure 1.**
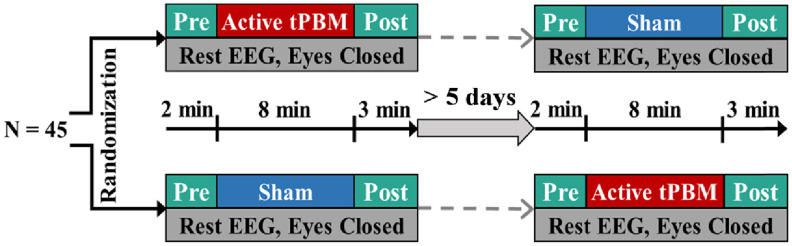
A crossover experimental protocol for tPBM and sham experiments (*n* = 45) with simultaneous EEG recording. The participants were at wakeful resting state with eyes closed.

The stimulation protocol (figure 1) consisted of a 2 min baseline (pre), an 8 min stimulation (tPBM or sham), and a 3 min recovery (post) period. The EEG data were acquired at either 256 Hz or 512 Hz; all 512 Hz data were down-sampled to 256 Hz during data pre-processing. tPBM was delivered near electrodes FP2 and AF8, under either sham or active conditions. The study was conducted in a single-blind crossover design, with each subject completing both sham and active tPBM experiments in a random order, with a minimum five day interval between the two experiments.

### 2.3. Overview of data processing steps

**Figure 2.**
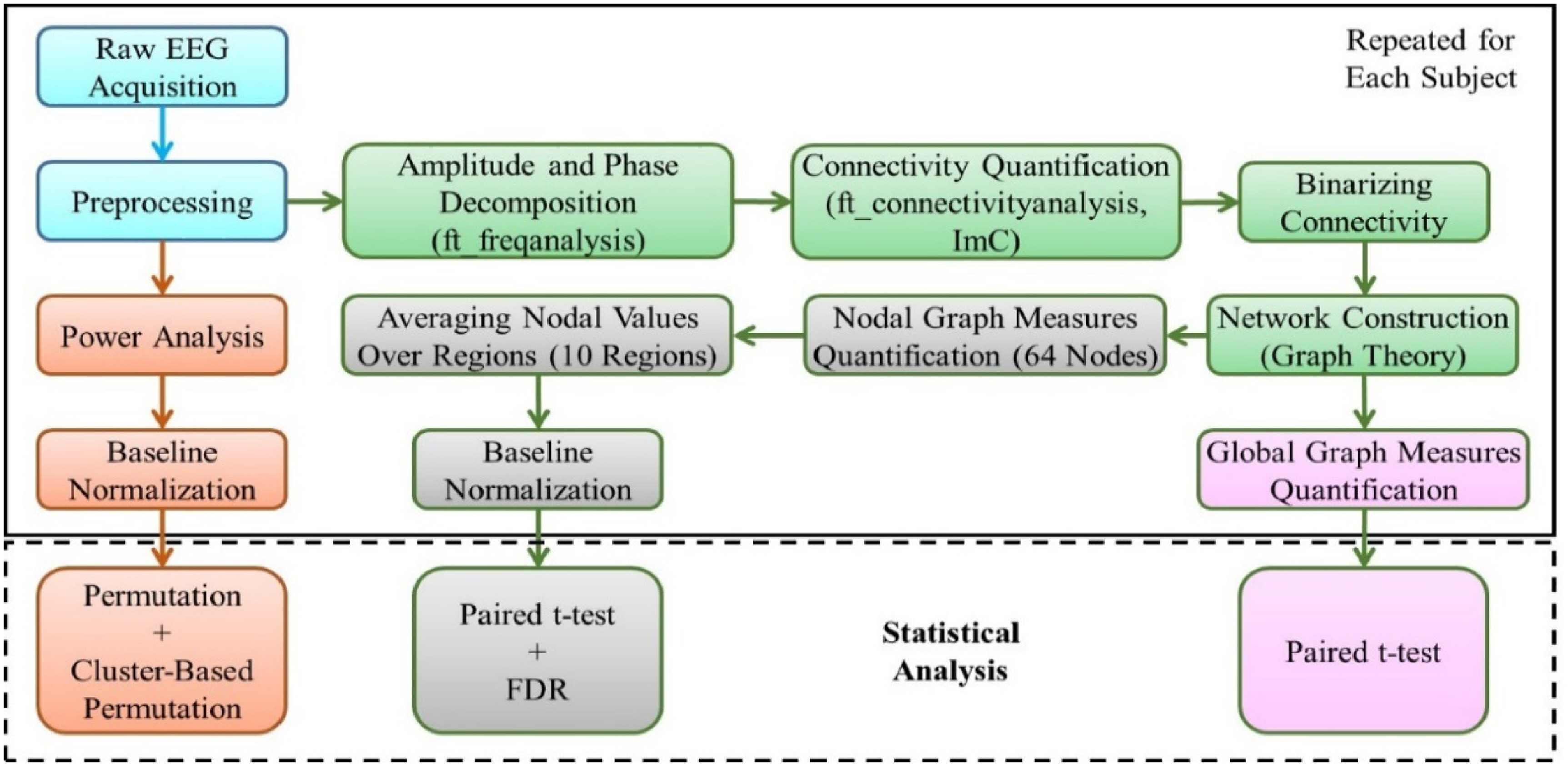
A data processing flow chart, including steps for (1) data pre-processing (blue boxes), (2) PSD-based analysis and permutation tests to form power topographies (orange boxes), (3) graphical edge formation based on the ‘imaginary part of coherence’ analysis (green boxes), (4) GTA-based assessment for global graphical connectivity metrics (pink boxes), and (5) GTA-based assessment for nodal graphical connectivity metrics (gray boxes).

Each EEG dataset represented a 13 min time series of 64 channels during both active and sham tPBM experiments from 45 participants. Because the data processing and analysis included multiple steps in this study, we outline a flow chart in figure 2 to guide the reader through them easily. The five subgroups in data processing are: (a) data pre-processing, (b) PSD-based analysis to obtain frequency-specific power topography, (c) graphical edge formation based on the ‘imaginary part of coherence’ analysis, (d) GTA-based quantification of global connectivity altered by tPBM, and (e) GTA-based analysis to identify local and nodal graphical metrics changed by tPBM.

### 2.4. Data pre-processing for EEG time series

EEGLAB (an open-source package) was used for data pre-processing. First, EEGLAB’s ‘filtfilt’ function was used to band-pass filter (0.5–70 Hz) the raw EEG data with zero phase distortion, followed by a 60 Hz notch filter to remove line noise. Next, each EEG series was re-referenced by the voltage averaged over all the 64 channels. Robust PCA was then applied to identify and remove significant signal artifacts and outliers from EEG signals [61, 62], followed by independent component analysis [63, 64] to further remove motion artifacts [65, 66], such as eye movements, saccades, and jaw clenching.

To quantify the dose-dependent responses of EEG to tPBM, each artifact-free time series was divided into four temporal sections: (a) the last minute of the 2 min baseline before the onset of stimulation (pre); (b) the first 4 min stimulation period (Stim1); (c) the second 4 min stimulation period (Stim2); and (d) the first 2 min recovery (post).

### 2.5. EEG PSD and changes in power

We used Welch’s method to quantify EEG PSD [67]. Specifically, with the use of the ‘Pwelch’ function (with a 4 sec window and 75% overlap [68]) in EEGLAB, a PSD curve of artifact-free time series for each EEG channel in each time section was calculated. Frequency-specific PSD bandwidths were then selected to cover the delta (1–4 Hz), theta (4–8 Hz), alpha (8–13 Hz), beta (13–30 Hz), and gamma (30–70 Hz) bands. Next, the mean power change at each of the five frequency bands (*f*), Δ*m*Power^f^, during each of the three temporal segments (Stim1, Stim2, and post) was normalized to the last minute of its baseline (pre), as expressed [38]:

\begin{align*} { }\Delta m{\text{Powe}}{{\text{r}}_i}^{\text{f}}&amp; = \frac{{{\text{PS}}{{\text{D}}^{\text{f}}}_i - {\text{PS}}{{\text{D}}^{\text{f}}}_{{\text{pre}}}}}{{{\text{PS}}{{\text{D}}^{\text{f}}}_{{\text{pre}}}}} \times 100\% \nonumber\\ &amp; = \frac{{\left( {{\text{PS}}{{\text{D}}^{\text{f}}}_i - {\text{PS}}{{\text{D}}^{\text{f}}}_{{\text{pre}}}} \right) \times {f_{{\text{band}}}}}}{{{\text{PS}}{{\text{D}}^{\text{f}}}_{{\text{pre}}} \times {f_{{\text{band}}}}}} \times 100\% \nonumber\\ &amp; = \frac{{{\text{Powe}}{{\text{r}}^{\text{f}}}_i - {\text{Powe}}{{\text{r}}^{\text{f}}}_{{\text{pre}}}}}{{{\text{Powe}}{{\text{r}}^{\text{f}}}_{{\text{pre}}}}} \times 100\% { } \end{align*}

where superscript ‘*f*’ denotes the five frequency bands, subscript ‘*i*’ represents the three temporal segments (Stim1, Stim2, or post), subscript ‘pre’ represents the baseline segment, *f*_band_ denotes the bandwidth of a chosen frequency band for PSD calculations, and PSD*_i_* and *PSD*_pre_ indicate bandwidth-averaged PSD values. Note that Δ*m*Power is a relative value or percentage change in the bandwidth-averaged power caused by tPBM or sham treatment (see the first two orange boxes in figure 2). To illustrate the difference in Δ*m*Power between the two conditions, we calculated the sham-subtracted (ss) and tPBM-induced change in power (Δ*m*Power_ss_) at each electrode for each of the five frequency bands within each of the three temporal periods:

\begin{align*}{ }\Delta m{\text{Power}}_{{\text{ss}},i}^{\text{f}} = \Delta m{\text{Power}}_{{\text{tPBM}},i}^{\text{f}} - \Delta m{\text{Power}}_{{\text{sham}},i}^{\text{f}}.\end{align*}

### 2.6. Statistical analysis for EEG power topography

Because EEG data at neighbouring time points and spatial channels are highly correlated, it is necessary to perform advanced statistical analysis to remove such correlations and for multi-variable comparisons. For this purpose, we utilized several functions (including ‘ft_freqstatistics’) available in the FieldTrip toolbox [69, 70] to perform CBPT for statistical comparisons of changes in EEG power (i.e. Δ*m*Power_ss_^f^ as shown in equation (2)) among the 64 electrodes in each of the five bands within each of the three temporal periods. In principle, CBPT has two components. The first is the cluster‐forming algorithm, which converts one high-dimensional observation into a quantifiable summary of the cluster structure. The second one creates a surrogate null distribution, against which the observed data are compared to obtain *p*-values [71].

Accordingly, we first grouped electrodes as clusters within a given scalp distance (e.g. 4–5 cm), followed by the identification of the EEG channels whose Δ*m*Power_ss_^f^ values were significantly different from zero for each electrode at a significance level of 0.05. Second, statistical evaluation was performed by taking the sum of the t-values for each cluster. Third, the summed *t*-value was compared with a null distribution. The null distribution for both permutation tests and cluster-based correction was obtained by randomly permuting the Δ*m*Power_ss_^f^ values 1000 times (see the last orange box in figure 2). The corresponding brain regions with modulated powers were identified in this way.

### 2.7. Amplitude and phase decomposition of EEG signal

For GTA-based connectivity quantification, we determined the edges or links of a graphical network between all pairs of EEG electrodes. Since correlations between the phases or amplitudes of these EEG channels are interpreted as functional connectivity between these points [41, 51], we performed amplitude and phase decompositions of the time series for all the 64 channels. The amplitude and phase of an EEG time-point can be represented as a complex number [41, 52]. Moreover, we utilized multiple tapers, namely, Slepian sequences, to taper the EEG signal in the time domain before performing the Fourier transform [72, 73]. This part of the calculation was conducted using the ‘ft_freqanalysis’ function within the FieldTrip toolbox [69].

### 2.8. Imaginary part of coherence as connectivity measure

Coherence, a widely used connectivity measure, is a frequency-domain function equivalent to the time-domain cross-correlation function. The coherence coefficient is a normalized quantity between 0 and 1 and is computed mathematically for the frequency of *ω* as follows [41]:

\begin{equation*}co{h_{xy}}\left( \omega \right) = \frac{{\left| {{S_{xy}}\left( \omega \right)} \right|}}{{\sqrt {{S_{xx}}\left( \omega \right){S_{yy}}\left( \omega \right)} }},\end{equation*}

where *S_xx_* and *S_yy_* denote the power estimates of the signals *x* and *y*, respectively, and *S_xy_* represents the averaged cross-spectral density term of the two signals. These terms were calculated using complex values obtained using the multitaper method.

Because of volume conduction in the human brain, signals generated in one region can be detected by several electrodes, resulting in an artificially high coherence value among these channels. To overcome this effect, the magnitude of the operation can be removed from equation (3), the imaginary part of *S_xy_* is considered, and the cross-spectral density of the signals with a phase difference of 0 or 2*π* is set to zero. This method is called the ‘imaginary part of coherence’, which explicitly removes instantaneous interactions [41]. Studies have shown that this method is excellent to minimize the volume conduction issue in EEG data analysis [74].

The pairwise connectivity values for all pairs of electrodes (64 in this study) can be represented by an *n* × *n* (i.e. 64 × 64) adjacency matrix, where n is the number of nodes (i.e. 64 channels). The FieldTrip toolbox facilitates the computation of the imaginary part of coherence for all pairs of channels using the ‘ft_connectivityanalysis’ (the first two green boxes in figure 2).

In this study, each temporal segment was divided into 10 sec epochs, and the adjacency matrices generated for all epochs in each frequency band were averaged for each of the three temporal segments and five frequency bands for each subject individually. These averaged matrices were binarized by varying the sparsity level and used for GTA-based global and nodal connectivity analyses, as described below.

### 2.9. Global and nodal graphical metrics selected for GTA

The GTA enables the exploration of topological changes in brain networks through pairwise functional connectivity between channels. A network can be characterized based on functional segregation, functional integration, and centrality. Previous studies showed tPBM-induced alterations in graph measures of the brain network with different setups and protocols [36, 40]. However, these studies focused on residual modulation in post stimulation with 19 channels.

In this study, we used GRETNA [75], a widely used GTA toolbox, to quantify the global and nodal graphical metrics of the human brain network for individual subjects under active and sham tPBM in four temporal segments and five frequency bands. This step was repeated 19 times to assess the chosen metrics under a sparsity range of 5%–95% with an increment of 5% (see the last two green boxes in figure 2).

To examine tPBM-induced effects, three global graphical measures were chosen for analysis: synchronization (S), global efficiency (GE), and small-worldness (SW). The group-level values for each global measure at each sparsity level were statistically compared between the active tPBM and sham conditions using a paired t-test (pink boxes in figure 2).

Two previous publications showed alterations only in global network metrics [40] and within each hemisphere or between two hemispheres [36]. In this study, our analysis quantified five nodal graphical metrics, namely, nodal clustering coefficient (nCC), nodal efficiency (nE), nodal local efficiency (nLE), betweenness centrality (BC), and degree centrality (DC). The respective definitions of graph metrics are listed in supplementary material A.

### 2.10. Topographical clusters for nodal connectivity

**Figure 3.**
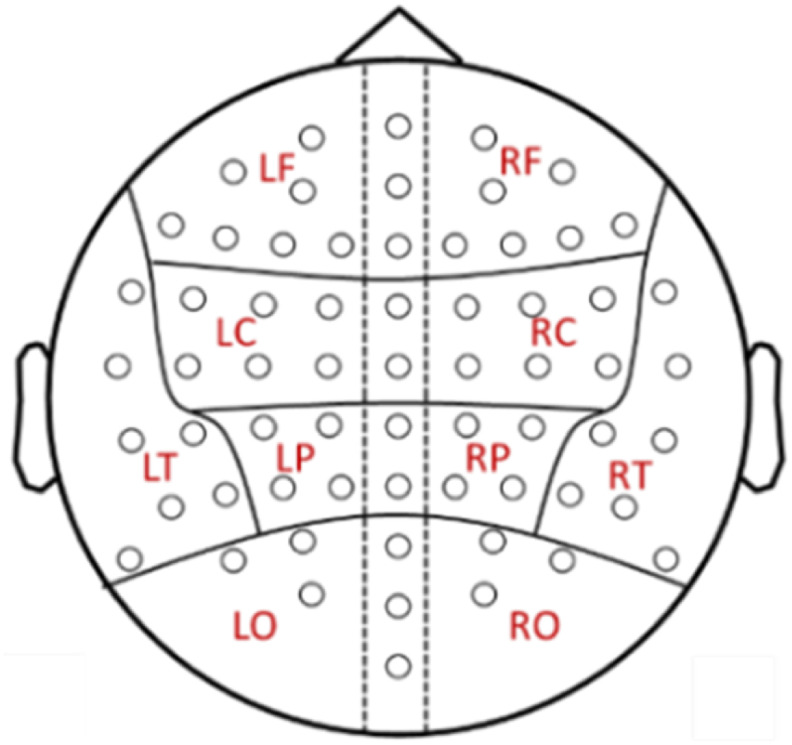
A layout of ten clusters for the 64 EEG electrodes. Circles represent electrodes; lines separate clusters. The ten medial electrodes are grouped twice in left and right regions. The overlapped regions are marked by the two dashed lines. LF: Left frontal; RF: Right frontal; LC: Left central; RC: Right central; LP: Left parietal; RP: Right parietal; LT: Left temporal; RT: Right temporal; LO: Left occipital; RO: Right occipital.

Although GTA was performed on the 64-channel EEG, resulting in tPBM-induced changes in nodal network metrics, the 64 nodal locations were too dispersed to identify the cortical regions on the human scalp. Thus, we focused on ten local sections according to prefrontal, central, temporal, parietal, and occipital regions [76]. Thus, 64 nodes were grouped into ten clusters with 6–10 electrodes in each cluster (figure 3).

At the subject level, each nodal graphical metric within each cluster area was obtained by averaging the specific metric over all the electrodes within the respective region (for each of the three temporal segments and five frequency bands). To compare the changes induced by tPBM and sham, nodal measures for each temporal segment were baseline-normalized by subtracting the corresponding baseline (pre) values from those in each of the three time windows (Stim1, Stim2, and post). Next, for each cluster region, group-level (*n* = 45) and baseline-subtracted nodal metric values were compared between the active and sham conditions using paired t tests. To correct for multiple comparisons, false discovery rate (FDR) correction was performed for ten regions with a corrected significance level of 0.05 (see the two vertical gray boxes in figure 2).

## 3. Results

### 3.1. Topographic changes in EEG power between tPBM and sham stimulations

**Figure 4.**
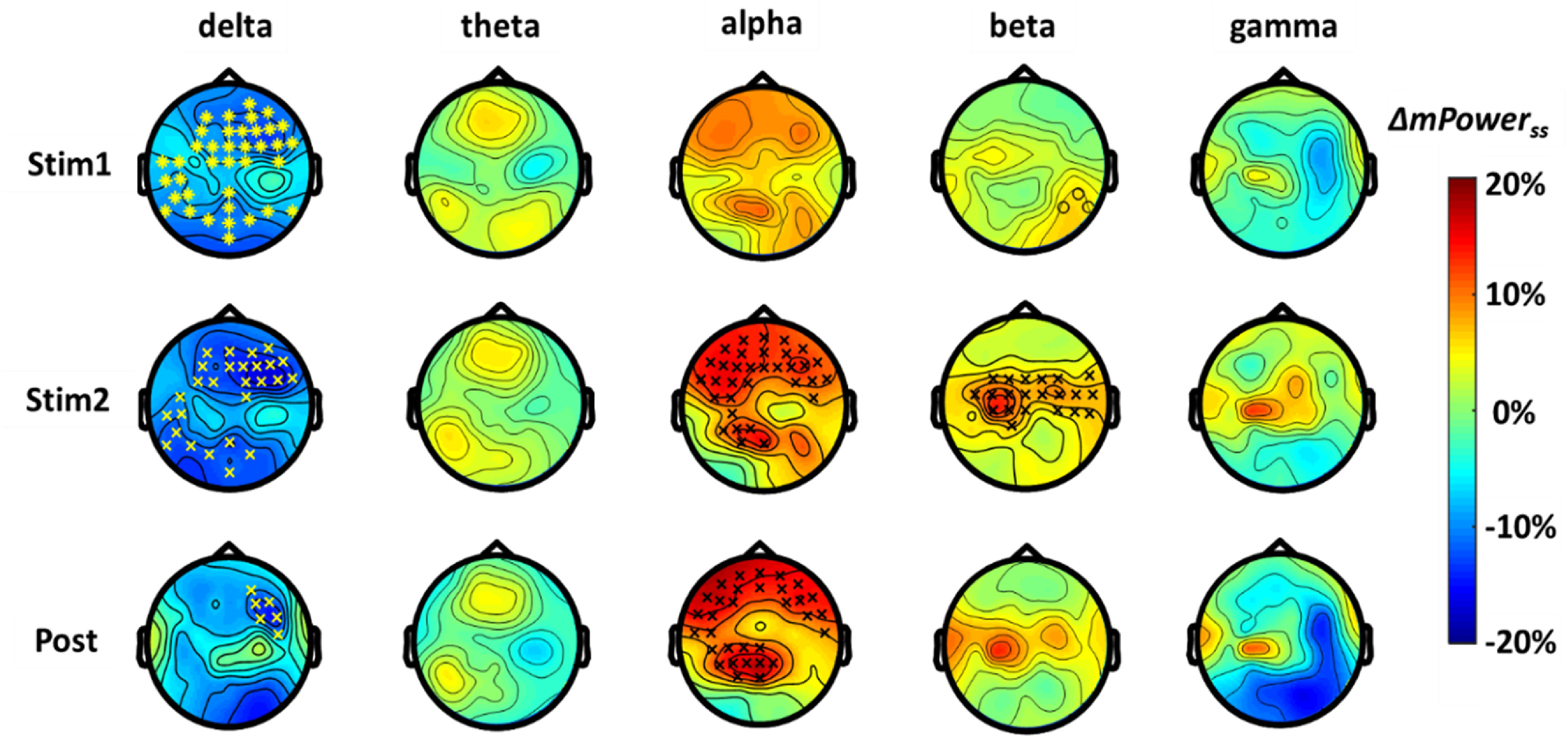
Topographic maps of group-averaged (*n* = 45), baseline-normalized, and sham-subtracted changes in Δ*m*Power_ss_ (see equation (2)) in delta (1–4 Hz), theta (4–8 Hz), alpha (8–13 Hz), beta (13–30 Hz), and gamma (30–70 Hz) bands during the first 4 min of tPBM (Stim1), second 4 min of tPBM (Stim2), and post tPBM period. Also, statistical results after the cluster-based permutation testing are superimposed in each topographical map, showing significant differences in Δ*m*Power between the tPBM and sham stimulations during respective three time segments and in five frequency bands with corrected significance levels of *p* < 0.05 (×) and *p* < 0.01 (*).

As described in sections 2.5 and 2.6, the baseline-normalized values of Δ*m*Power^f^ (see equation (1)) for each group of tPBM and sham conditions among the three temporal segments (Stim1, Stim2, and post) and in five frequency bands were calculated. To demonstrate clear statistical differences in Δ*m*Power^f^ between the two stimulation conditions, baseline-normalized and sham-subtracted topographical maps of Δ*m*Power_ss_^f^ (%) values (see equation (2)) over 64 channels were achieved, as shown in figure 4, for all five frequency bands. In addition, after CBPT for 64-channel statistical comparison, the electrode sites/clusters that were significantly affected by tPBM are superimposed on the topographies in figure 4 with ‘*’ denoting *p* < 0.01 and ‘×’ denoting *p* < 0.05.

These results illustrate a significant, dose-dependent increase in EEG rhythm powers at 8–13 Hz and 13–30 Hz during the last 4 min of tPBM (Stim2). Specifically, the increase in alpha Δ*m*Power_ss_ was seen as two major clusters of channels in the bilateral frontal and left parietal-occipital regions, whereas the increase in beta Δ*m*Power_ss_ was mainly seen as one cluster of electrodes in the central/parietal region of the scalp. The enhanced alpha Δ*m*Power_ss_ remained in the affected locations during the post-tPBM period, whereas the significant increase in beta Δ*m*Power_ss_ ceased during the recovery time. Furthermore, delta power was reduced in the frontal, left temporal, and occipital regions during tPBM, and in the right frontal region during recovery.

### 3.2. Global graphical metrics of functional connectivity altered by tPBM

**Figure 5.**
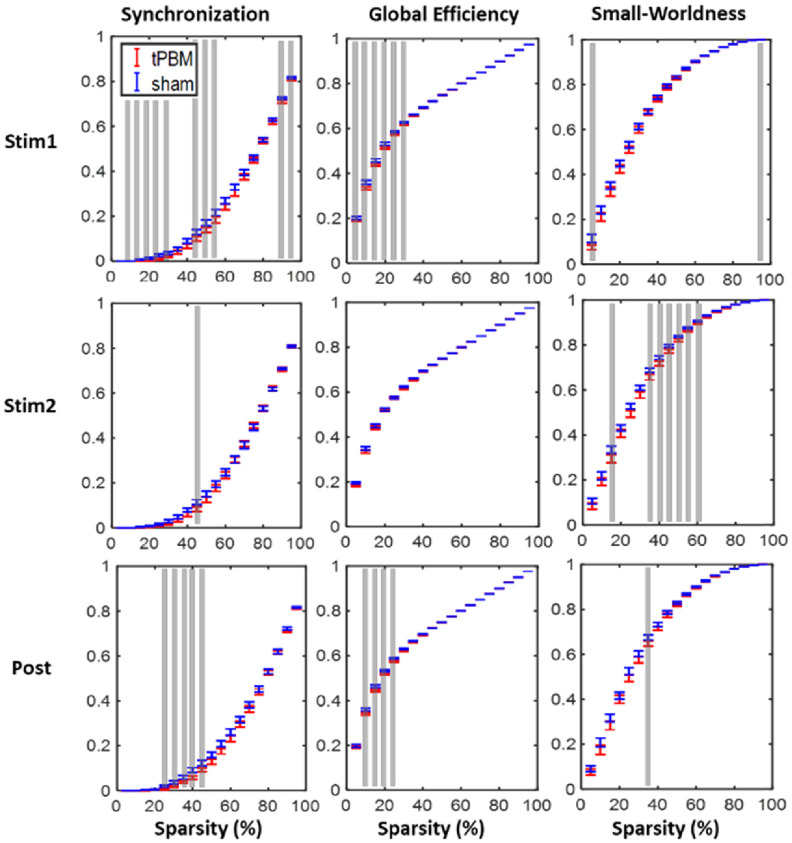
Three GTA-derived global graphical metrics, namely, synchronization (the 1st column), global efficiency (the 2nd column), and small-worldness (the 3rd column), of the EEG brain network in the beta band (13–30 Hz) under both active tPBM and sham stimulation during Stim1 (the 1st row), Stim2 (the 2nd row), and the post period (the 3rd row). In each panel, the *y* axis denotes respective metric values while the *x* axis presents sparsity values with an increment of 5%. The grey bars mark sparsity values at which the corresponding graphical metrics were altered significantly by tPBM with respect to sham based on paired t-tests (*p* < 0.05).

Following the steps given in sections 2.7–2.9, adjacency matrices for all three temporal segments and five frequency bands were generated. These matrices were further binarized for different sparsity values, resulting in the GTA-derived graphical networks. In this study, we identified three global network metrics (S, GE, and SW) that were significantly altered by tPBM with respect to the sham condition and only in the beta band. As shown in figure 5, the three rows illustrate the respective global metrics during Stim1, Stim2, and the post period under both active and sham stimulation.

These results clearly show that tPBM significantly reduces the global synchronization, GE, and SW of the network connectivity of the human brain. Specifically, significant decreases in synchronization and GE occurred during Stim1 and the recovery period with more sparsity values, while a significant reduction in SW appeared in Stim2 with more sparsity units. We also confirmed that there was no significant difference between the pre-stimulation baselines under tPBM and sham conditions for any of the three global network metrics.

### 3.3. Nodal graphical metrics of functional connectivity altered by tPBM

**Figure 6.**
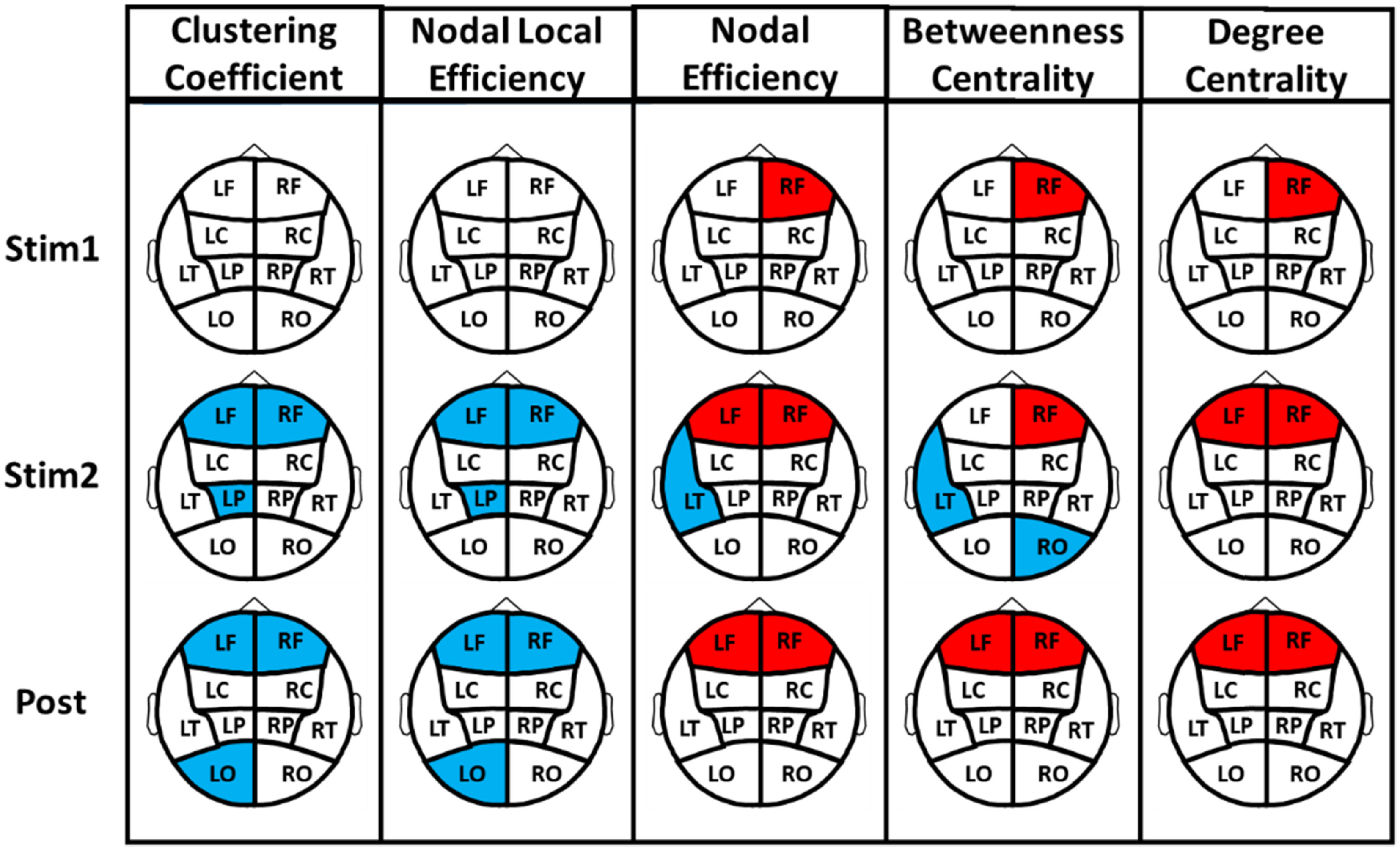
Comparative topographical maps of ten-Cluster-distributed nodal network metrics at the beta band (13–30 Hz). The comparison was made between tPBM and sham stimulation conditions for each of the five nodal metrics, namely, the clustering coefficient (the 1st column), nodal local efficiency (the 2nd column), nodal efficiency (the 3rd column), betweenness centrality (the 4th column), and degree centrality (the 5th column) of the EEG brain network during Stim1 (the 1st row), Stim2 (the 2nd row), and the post period (the 3rd row). LF: Left frontal, RF: Right frontal, LC: Left central, RC: Right central, LP: Left parietal, RP: Right parietal, LT: Left temporal, RT: Right temporal, LO: Left occipital, RO: Right occipital. Red color represents tPBM > sham; blue color indicates tPBM < sham with *p* < 0.05 (FDR corrected).

After performing the analysis steps given in sections 2.9 and 2.10, cluster-averaged, baseline-subtracted values for each of the five nodal graphical metrics (i.e. nCC, nE, nLE, BC, and DC) were obtained for each of the ten spatial clusters under both tPBM and sham conditions. After performing paired t-tests with FDR correction for ten spatial clusters (i.e. *p* < 0.05, FDR corrected), we identified the clusters whose nodal metric values were significantly altered by tPBM for each of the five metrics at all three temporal periods and only in the beta rhythm band. Topographical representations of the results for the five nodal metrics are shown in figure 6.

Based on figure 6, we made the following observations. (a) During Stim1, significant increases in nE, BC, and DC were observed in the right frontal region near the tPBM stimulation site. (b) During Stim2 and post stimulation, significant changes occurred in the bilateral frontal regions for all five nodal metrics. More specifically, tPBM significantly decreased the clustering coefficient and nLE, whereas stimulation significantly increased the other three nodal metrics. (c) Combined temporal and spatial results revealed that both nE and DC were initially enhanced by tPBM in the right frontal region during Stim1, followed by expansion of this enhancement to the contralateral side during Stim2, which persisted during the post-tPBM period. (d) In the case of BC, unilateral enhancement in the right frontal region remained during the entire stimulation time (Stim1 and Stim2) and then expanded to the contralateral side in the post. (e) On the other hand, during Stim2 and post-stimulation, significant decreases occurred in the bilateral frontal regions for the nCC and nLE. (f) During the same time periods for the same two metrics (nCC and nLE), decreases were observed in the left parietal and occipital regions. (g) Moreover, the left temporal region showed a reduction in nE and BC only for Stim2. (h) The only significant modulation in the right occipital region was a decrease in BC in Stim2.

## 4. Discussion

In section 3, we describe the clusters and regions on the scalp where tPBM modulates EEG oscillation powers and GTA-based EEG beta network connectivity. In this section, we will interpret our observations, compare our results with previous studies, and associate the neurophysiological changes in different brain regions with behavioral improvement by tPBM that has been reported by others [3, 11, 13–17, 57].

### 4.1. tPBM-induced alterations on EEG Δm Power in clusters of electrodes

As shown in figure 4, under the eyes-closed resting-state condition, tPBM significantly increased the power of alpha oscillations in clusters over the bilateral frontal, left parietal, and left occipital regions, as well as the beta power over the bilateral central and parietal regions during the second 4 min of stimulation. These observations agree with previous reports on eyes-open tPBM experiments [60, 77].

A significant increase in alpha power over the frontal-parietal regions clearly confirmed the ability of tPBM to neuromodulate the frontoparietal network, which is an executive network facilitating rapid instantiation of new tasks [78]. It is reported that alpha rhythm is associated with awareness [79] and cognitive functions, such as memory encoding and attention [80–82]. The same experimental protocol with a 1064 nm laser was used previously and demonstrated a significant improvement in human cognitive performance [3, 13–15, 57]. Moreover, the presence of stronger beta waves has been linked to better cognitive ability, as reported in several studies [83, 84]. Thus, the beneficial outcome of frontal tPBM in human cognition can be, at least partially, attributed to its significant modulation of electrophysiological alpha and beta powers in the frontoparietal network. In addition, we attributed the reduction in delta power in the channel clusters during tPBM to the sensation of laser-induced warmness in the forehead skin [38].

### 4.2. tPBM-induced alterations in global measures of functional network in beta band

As shown in figure 5, tPBM significantly changed three global graph measures, namely, synchronization, GE, and SW, in the beta wave only. We discuss each of these changes as follows.

Our observation that brain network synchronization in the beta band was significantly reduced during Stim1 and recovery agrees with a recent study that reported the effects of tPBM on brain network synchronization with 850 nm LEDs [36]. In addition, a behavioral study attributed a decrease in synchronization in the healthy human brain to awareness and cognitive processing [85].

Similarly, the GE was significantly reduced by tPBM compared with sham during Stim1 and post. This reduction indicates a decrease in brain network integration, which may imply less efficient or more complex information paths in the network. In other words, tPBM may increase the energy and wiring costs of information flow owing to the trade-off between network efficiency, energy, and wiring costs [86, 87]. This could be an indicator of increased brain complexity related to higher cognitive function [88]. This observation supports the expected benefit of tPBM, namely, the beneficial effects of tPBM on cognitive improvement.

SW is calculated as the ratio of normalized integration to normalized segregation [50]. Thus, the reduction in this metric could be attributed to a significant reduction in global integration (as reflected by a reduction in GE) and/or a significant increase in global segregation of the brain network caused by tPBM. These observed significant effects of tPBM on SW taking place only in Stim2 could result from the resistance of resting-state networks against changes in network composition as well as the dose-dependent nature of tPBM-induced effects on neural activity [89, 90]. However, a possible explanation for the lack of significant alterations in synchronization and GE in Stim2 could be the high variability in the functional topography of the frontoparietal network [78].

In addition, the observation that tPBM altered only the beta-wave oscillation in the EEG graphical network was in agreement with other studies [36, 49]. Several studies have shown the role of the beta band in different brain networks [91] and cognitive functions [92, 93].

### 4.3. tPBM-induced alterations in nodal graphical measures in beta band

Significant reductions in nCC and nLE in bilateral frontal regions and in the left parietal and left occipital regions during Stim2 and post implied that the clusters of nodes in these regions of the network became less segregated during the 2nd 4 min and post period of tPBM. In other words, tPBM facilitated less separation and more integration of nodal graphical connectivity. Consistently, nE (reflecting nodal integration) increased in the right frontal region in the beginning of tPBM. This increase indicates enhanced integrity of the nodes over this region in the information flow [94] and parallel information transfer. Furthermore, during the last 4 min of tPBM, the bilateral frontal regions showed an increase in integration into the functional network, while the integrity of the left temporal region in the network was significantly reduced. This phenomenon implies that tPBM stimulated more network integration at the beta rhythm in the frontal regions, with the cost of reducing network integration in the left temporal segment/cluster.

BC represents the fraction of all shortest paths in the network that pass through a particular central node [95]. A large value of BC means a large impact of this central node on information flow over the network. Also, DC quantifies the number of links from nodes in a specific region to other nodes in the same or other regions [50]. Increases in both BC and DC in the frontal region during and post tPBM suggested that the frontal regions became more prominent in connecting disparate parts of the network. The enhancement of BC and DC revealed that these cortical regions could be prominently stimulated by tPBM for more information connections at beta oscillations. It has also been reported that a working memory task during the encoding phase triggers similar increases in DC over the frontal regions of the beta band [93].

Combining all these observations, we concluded that tPBM facilitated a reduction in local segregation, increases in nodal integration and centrality of frontal regions, and the growth of connection links between nodes in these frontal regions compared to other regions. These results agree with the observed changes in the flow of information reported in a tPBM-evoked causal connectivity study [96].

### 4.4. The role of the beta band in tPBM-induced network modulation

The alpha and beta powers of the human brain, especially in the frontoparietal network, are believed to be related to cognitive functions, such as memory encoding and attention [80–82]. Our observations clearly demonstrated that tPBM increased the alpha and beta powers in the frontal-central-parietal regions, indicating the underlying association between tPBM and the enhancement of human memory.

However, our results showed that tPBM altered EEG graphical network metrics only in the beta band, which was consistent with the results given in [36]. According to [91], beta oscillations in the prefrontal region appear to serve as short-term memory executors and focus enhancers during executable tasks. In addition, the beta rhythm in the temporal lobe plays an important role in long-term memory retrieval [97]. Memory retrieval starts in the temporal lobe, passes through different parts of the neocortex, and stops in the prefrontal cortex [91]. There is always a balance in the information flow in this order, even during the resting state. We speculated that tPBM enables neuromodulation of beta oscillations and the corresponding network connectivity globally across the scalp and regionally in several nodal regions. The significant modulation of beta-wave connectivity in the human brain may be an underlying association between tPBM and the enhancement of human cognition. This speculation may be validated through PET imaging using fluorodeoxyglucose [98].

### 4.5. Comparisons to two other publications

As shown in table 1, only two recent studies reported tPBM-induced modulations of global network metrics [40] and alterations in brain connectivity between the two hemispheres [36]. It would be helpful to compare the results from the two studies with those found in this study. Detailed summaries and comparisons are given in supplementary material B.

### 4.6. Two opinions on basic mechanism of tPBM

There is a relatively unresolved debate regarding the basic mechanism of action of tPBM. One side of the debate states that a red or NIR photon can photo-dissociate nitric oxide (NO), a molecule that may inhibit CCO by noncovalent binding [99, 100]. This view emphasizes the greater effects of tPBM on diseased/damaged cells because unhealthy cells are more likely to have inhibitory concentrations of NO. On the other hand, the other opinion highlights tPBM-induced effects on CCO, which stimulates ATP production by increasing mitochondrial membrane potential and oxygen consumption [26, 58]. This view enables to explain the tPBM-induced augmentation of human cognition in healthy humans besides in patients with brain disorders [26, 58, 101]. This side of opinions has been supported by several publications [58], and now by two other independent studies that also reported significant improvement of working memory in healthy young [102] and older adults [103] by repeated tPBM. However, the two opinions on the basic mechanism of tPBM have coexisted for years, without one outweighing the other. Further confirmation of each remains to be explored in future studies.

### 4.7. Limitations and future work

First, the international 10–10 electrode placement system in this study was not strictly followed on the human head because a clear area with a 4.2 cm diameter was needed for tPBM light delivery. The EEG cap was shifted 1–2 cm backwards. Second, our power spectral and connectivity analyses were performed in the sensor space. Source space analysis can be conducted to observe cortical and subcortical regions in the brain that are affected by tPBM. Third, the current study was based on EEG signals of the tPBM-treated human brain in the resting state. Concurrent assessments of changes in cognitive enhancement and brain connectivity after tPBM would provide quantitative correlation and association between functional connectivity and behavioral effects of tPBM.

As for future work, there is a lack of systematic studies on the optimization parameters of tPBM, such as the wavelengths used, light irradiance, stimulation dose, and sites, as well as the effective period after stimulation. In particular, tPBM has a hormetic dose response, which is characterized by stimulation of a biological process at a low dose and inhibition of that process at a high dose [26, 58]. Namely, photo-stimulatory or photo-inhibitory effects occur with low (0.001–10 J cm^−2^) and high (>10 J cm^−2^) optical energy (dose) density [104–106]. However, the documented dose range for positive responses results from *in vitro* experiments and is quite broad (0.001–10 J cm^−2^). It is difficult to select an optimal irradiance for non-invasive human treatments with tPBM. Accordingly, more research is needed with both healthy humans and selected populations of patients before tPBM becomes an effective device to treat patients with brain disorders and for healthy aging in the rapidly growing aging population.

## 5. Conclusion

In this study, we utilized three analytical steps to identify the electrophysiological effects of tPBM in a healthy human brain. First, power spectral analysis revealed that alterations in EEG spectral power were mainly present in the alpha and beta bands of the fronto-central-parietal regions. Second, a topological approach, GTA, facilitated findings on significant modulation of the EEG beta rhythm in the information path and enhancement of the brain network complexity at the global network level during and after the stimulation. Finally, assessment of the nodal measures of the network at the regional and cluster levels confirmed that tPBM had a major effect on frontal and parietal clusters in the beta band. The information paths were enhanced during and post tPBM in prefrontal regions near the stimulation site. Further studies are needed to better understand the relationship between tPBM-induced alteration of brain networks and improvement in human cognition if tPBM is to be developed as a useful tool for treating patients with brain disorders and supporting healthy aging in the aging population worldwide.

## Data Availability

The data that support the findings of this study are available upon reasonable request from the authors.
